# PNA lectin for purifying mouse acinar cells from the inflamed pancreas

**DOI:** 10.1038/srep21127

**Published:** 2016-02-17

**Authors:** Xiangwei Xiao, Shane Fischbach, Joseph Fusco, Ray Zimmerman, Zewen Song, Philip Nebres, David Matthew Ricks, Krishna Prasadan, Chiyo Shiota, Sohail Z. Husain, George K. Gittes

**Affiliations:** 1 Division of Pediatric Surgery, Children’s Hospital of Pittsburgh, University of Pittsburgh School of Medicine, 4401 Penn Ave, Pittsburgh, PA15224, USA; 2 Division of Pediatrics, Children’s Hospital of Pittsburgh, University of Pittsburgh School of Medicine, 4401 Penn Ave, Pittsburgh, PA15224, USA

## Abstract

Better methods for purifying human or mouse acinar cells without the need for genetic modification are needed. Such techniques would be advantageous for the specific study of certain mechanisms, such as acinar-to-beta-cell reprogramming and pancreatitis. Ulex Europaeus Agglutinin I (UEA-I) lectin has been used to label and isolate acinar cells from the pancreas. However, the purity of the UEA-I-positive cell fraction has not been fully evaluated. Here, we screened 20 widely used lectins for their binding specificity for major pancreatic cell types, and found that UEA-I and Peanut agglutinin (PNA) have a specific affinity for acinar cells in the mouse pancreas, with minimal affinity for other major pancreatic cell types including endocrine cells, duct cells and endothelial cells. Moreover, PNA-purified acinar cells were less contaminated with mesenchymal and inflammatory cells, compared to UEA-I purified acinar cells. Thus, UEA-I and PNA appear to be excellent lectins for pancreatic acinar cell purification. PNA may be a better choice in situations where mesenchymal cells or inflammatory cells are significantly increased in the pancreas, such as type 1 diabetes, pancreatitis and pancreatic cancer.

The utility of islet transplantation for type 1 and some cases of type 2 diabetes has been limited by a shortage of donor pancreases^1^,^2^,^3^. Postnatal beta-cell mass expansion is predominantly derived from beta-cell replication^4^,^5^,^6^,^7^,^8^,^9^,^10^. However, beta-cell replication may be slow, and likely decreases progressively with age^11^,^12^,^13^,^14^,^15^,^16^,^17^. Thus, great efforts have been made to generate functional beta-cells from non-beta cell sources. Acinar cells are the most abundant cell type in the human and mouse pancreas, a characteristic that makes them an attractive potential source of new beta-cells. Indeed, some previous work has supported this possibility *in vivo*^18^,^19^,^20^, and *in vitro*^21^,^22^.

In order to optimize acinar-to-beta cell reprogramming, *in vitro* testing of potential strategies is necessary. This *in vitro* testing requires an optimal technique for purifying acinar cells from the pancreas^8^,^21^,^22^,^23^,^24^. In particular, the reliable elimination of pre-existing beta cells from acinar cell preparations is critical here, but can be challenging due to the presence of beta-cell degranulation^25^,^26^,^27^ and dedifferentiation^28^,^29^,^30^,^31^, making the beta-cells difficult to immune-detect. Hence, correctly purifying acinar cells is an indispensable step towards clinical acinar-to-beta cell conversion.

Isolation of acinar cells using genetically modified mice in which a fluorescent reporter is expressed under an acinar-cell-specific promoter has its advantages and disadvantages. For example, we have previously used a tamoxifen-treated elastase-CreERT; ROSA26^Tomato^ (Ela-Cre; TOM) mouse model to isolate acinar cells based on red fluorescence by flow cytometry, and have shown that the purified acinar cells lack contaminating non-acinar pancreatic cells, such as synaptophysin (SYN)-positive endocrine cells, CD31-positive endothelial cells, Dolichos biflorus agglutinin (DBA)-positive duct cells, vimentin-positive mesenchymal cells and CD45-positive inflammatory cells^32^,^33^,^34^. However, these mice could not be used in studies in which acinar cells need to be isolated from mice with other Cre-mediated genetic modifications, e.g. gene ablation or overexpression. Similarly, such genetic labeling strategies are not realistic for human pancreas. Therefore, purification of acinar cells through non-genetic approaches is strongly preferable.

Earlier studies have utilized lectin affinity to acinar cells to purify acinar cells by flow cytometry^21^,^35^. These investigations provide a strong basis for establishing a technology for purifying human and mouse acinar cells without acinar-cell-specific genetic labeling. However, the efficiency and quality of the purification was not evaluated in these previous studies, where only major cell types (beta cells, duct cells and acinar cells) were checked for lectin-affinity^21^. Here, in this study, we aimed to assess the binding affinity and specificity of different lectins to acinar cells in order to identify the lectin that optimizes acinar cell purification.

## Materials and Methods

### Protocol approval

All the mouse experimental methods in the current study were approved by the Animal Research and Care Committee at the Children’s Hospital of Pittsburgh and the University of Pittsburgh IACUC (Protocol number: 14031989). All the experiments have been carried out in accordance with the guidelines from the research committee at the Children’s Hospital of Pittsburgh and the University of Pittsburgh.

### Mouse strains

C57BL/6 mice and non-obese diabetes (NOD) mice were purchased from Jackson Lab (Bar Harbor, ME, USA). Bacterial-artificial-chromosome (BAC) transgenic elastase-CreERT; ROSA26^Tomato^ (Ela-Cre; TOM) has been described before^32^,^33^,^34^. Only female mice of each strain were analyzed at 12–16 week-old in the current study. To induce tomato (TOM) expression in acinar cells in Ela-Cre; TOM mice, 1 week before analyses, mice were given a single intraperitoneal injection of 1 mg tamoxifen in 50 μl corn oil, resulting in nearly 100% labeling of acinar cells without detectable non-specific labeling of other cell types^33^.

### Pancreatic digestion and fluorescence-activated cell sorting (FACS)

Pancreatic duct perfusion and subsequent complete digestion of the pancreas were performed with 0.25 mg/ml collagenase (Sigma-Aldrich, St. Louis, MO, USA) for 50 minutes to obtain a single cell population, as has been described before^8^,^34^,^36^. Then the Fluorescein-conjugated lectins (Catalog number: FLK2100, FLK3100, FLK4100, Vector Labs, Burlingame, CA, USA) or PE-cy7-conjugated rat-anti-mouse CD45 antibody (Becton-Dickinson Biosciences, San Jose, CA, USA) were incubated with the pancreatic digests at a concentration of 0.5 μl/ml on ice for 15 minutes before analyses by flow cytometry using a FACSAria (Becton-Dickinson Biosciences), as has been described before^8^,^32^,^34^. Purity of the sorted cells was determined by mRNA expression for amylase, CK19, CD31, synaptophysin (SYN), vimentin and CD45. For analysis of double-sorting, sequential sorting is routinely performed as a quality control. Flow cytometry data were analyzed and shown by FlowJo (Tree Star Inc, Ashland, OR, USA).

### Real-Time Quantitative Polymerase Chain Reaction (RT-qPCR)

RNA was extracted from digested pancreatic cells w/o FAC sorting using RNeasy (Qiagen, Valencia, CA, USA) and quantified with Nanodrop1000 (Thermo Fisher Scientific, Inc, Waltham, MA, USA) according to the manufacturer’s instructions, followed by cDNA synthesis (Qiagen). Primers were purchased from Qiagen for cyclophilinA (CypA; QT00247709), amylase (QT00179242), CK19 (QT00156667), CD31 (QT01052044), vimentin (QT00159670) and CD45 (QT00139405). RT-qPCR reactions were performed in duplicates with QuantiTect SYBR Green PCR Kit (Qiagen) using a LightCycler 1.5 Instrument (Roche, Branchburg, NJ, USA). Specificity of the amplified products was determined by melting point analysis. Quantification for each gene of interest was performed with the 2^−ΔΔCt^ method. Values for genes were first normalized against CypA, the housekeeping gene control, and then compared to the complete pancreatic digests, without sorting, as an experimental control.

### Immunostaining

Immunostaining and Western Blot analysis were performed as described before^32^,^33^,^36^,^37^,^38^. For immunostaining, briefly, the mouse pancreas was fixed and cryo-protected in 30% sucrose overnight before freezing. Fluorescein-conjugated Lectins were incubated with pancreas sections for 1 hour, and then washed 3 times with phosphate buffered saline (PBS), before detection of their binding to pancreatic tissue. As a control, PNA was pretreated with 200 mmol/l galactose for 15 minutes, before it was used for examination of tissue binding. DBA was detected after direct labeling and TOM was detected by direct fluorescence. Primary antibodies are: guinea pig polyclonal anti-insulin (Dako, Carpinteria, CA, USA), rabbit polyclonal anti-SYN (Invitrogen), and at polyclonal anti-CD31 and anti-CD45 (Becton-Dickinson Biosciences). Secondary antibodies were all purchased from Jackson ImmunoResearch Labs (West Grove, PA, USA). Nuclear staining was performed with Hoechst (Sigma-Aldrich). Staining and imaging of sections were performed as described previously^8^,^32^,^37^.

## Data Analysis

All values are depicted as mean ± standard error of the mean. Five mice were analyzed in each experimental group. All data were statistically analyzed using one-way ANOVA with a Bonferoni correction, followed by Fisher’s Exact Test for comparison between two groups, with a GraphPad Prism 6.0 (GraphPad Software, Inc. La Jolla, CA, USA).

## Results

### Two lectins appear to specifically bind to acinar cells in the mouse pancreas

**Figure 1:**
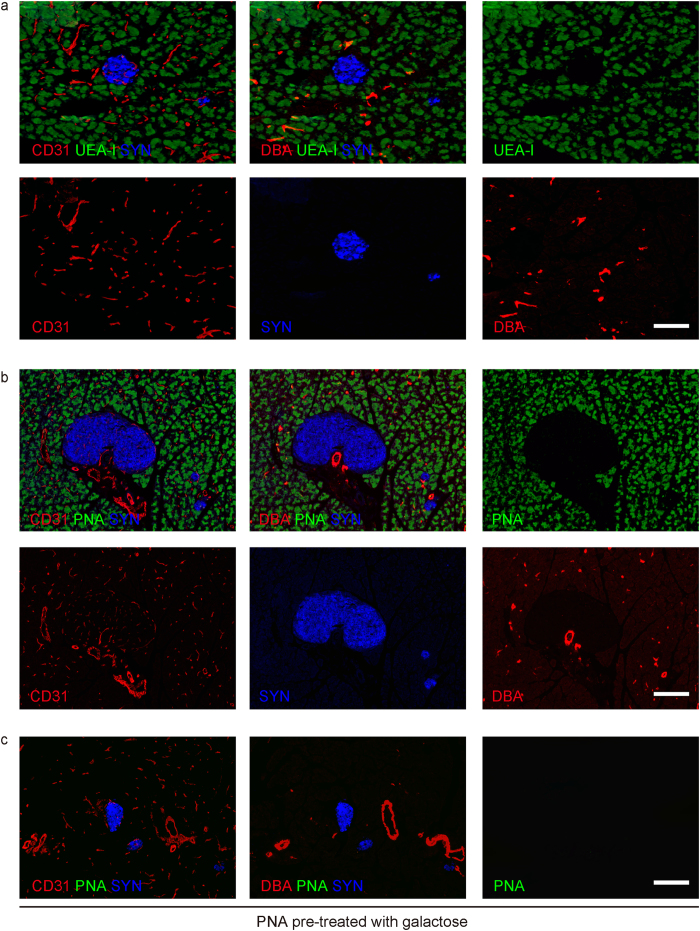
Two lectins appear to have specific affinity for acinar cells in the mouse pancreas. (**a,b**) C57/BL6 mouse pancreas was labeled with 2 specific lectins (UEA-I, a; and PNA, (**b**), and counterstained with synaptophysin (SYN), CD31, and DBA. (**c**) The PNA lectin was pre-treated with 200 mmol/l galactose for 15 minutes and then used to label C57/BL6 mouse pancreas prior to staining with SYN and CD31. Scale bars are 50 μm.

UEA-I lectin has been used to isolate acinar cells from the pancreas. Nevertheless, the exact purity of the acinar cell fraction has not been thoroughly evaluated. Here, we aimed to compare the binding affinity and specificity of different lectins for acinar cells in order to ascertain the optimal lectin for this purpose. First, we stained the mouse pancreas with 20 widely used FITC-conjugated lectins (Table 1), and found that 2 specific lectins [Ulex Europaeus Agglutinin I (UEA-I), Fig. 1a and Peanut agglutinin (PNA), Fig. 1b] bound strongly to acinar cells, while they appeared to have negligible binding to other major pancreatic cell types, including endocrine cells [by synaptophysin (SYN) staining], endothelial cells (by CD31 staining), and duct cells (by DBA staining). Further, pre-treatment with galactose appropriately completely blocked the binding of PNA lectin to acinar cells (Fig. 1c). Thus, these two lectins were chosen for further analysis.

### Purification of lectin-positive cells by flow cytometry

**Figure 2:**
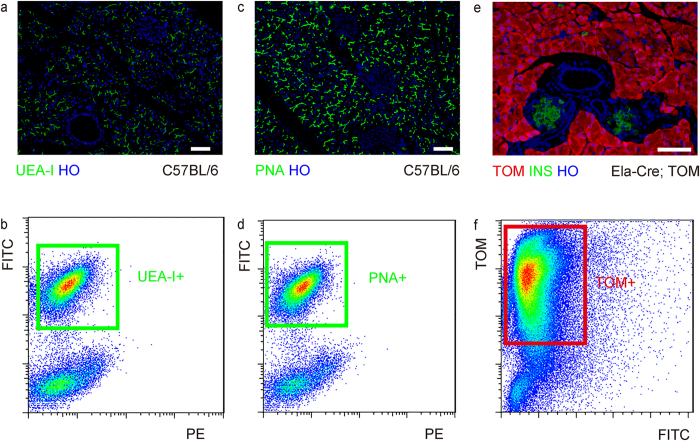
Purification of lectin-positive cells by flow cytometry. (**a–f**) Acinar cells were purified from either UEA-I-labeled C57BL/6 mouse pancreatic cells [representative immunohistochemistry control shown in (**a**)], based on green fluorescence by flow cytometry (**b**), or PNA-labeled C57BL/6 mouse pancreatic cells [representative immunohistochemistry control shown in (**c**)], based on green fluorescence by flow cytometry (**d**), or tamoxifen-treated Ela-Cre; TOM mouse pancreatic cells [representative tomato fluorescence control on sections shown in (**e**)], based on red fluorescence by flow cytometry (**f**). HO: Hoechst nuclear staining. Scale bars are 50 μm.

Apart from acinar cells, SYN-positive endocrine cells, CD31-positive endothelial cells and duct cells, there are still other cell types that represent minor populations in the pancreas, e.g. mesenchymal cells (positive for vimentin), and inflammatory cells (positive for CD45). However, these two minor populations can be significantly enriched in some pathological processes. For example, during pancreatitis and pancreatic fibrotic diseases, the number of mesenchymal cells dramatically increases, whereas during pancreatic inflammation (e.g. pancreatitis or autoimmune diabetes), the number of inflammatory cells present in the pancreas may be significantly increased. In these pathologic situations, the purity of acinar cells that are isolated based on lectin affinity could be significantly affected by the affinity of lectin to these other cell populations. Thus, we purified acinar cells from either UEA-I-labeled C57BL/6 mouse pancreas (Fig. 2a) based on green fluorescence (Fig. 2b), or PNA-labeled C57BL/6 mouse pancreas (Fig. 2c) based on green fluorescence (Fig. 2d), or tamoxifen-treated elastase-CreERT; ROSA^Tomato^ (Ela-Cre; TOM) mouse pancreas (Fig. 2e) based on red fluorescence (Fig. 2f). The purity of these different acinar cell preparations was compared.

### PNA-purified acina r cells contain fewer contaminating mesenchymal and inflammatory cells

The purity of sorted acinar cells based on UEA-I or PNA binding, or by tomato fluorescence in Ela-Cre; TOM mice was compared to the unlabeled dissociated pancreatic cells from C57BL/6 mice by examination of mRNA levels of specific cell marker genes, including amylase (for acinar cells), SYN (for endocrine cells), CK19 (for duct cells), CD31 (for endothelial cells), vimentin (for mesenchymal cells), and CD45 (for inflammatory cells).

For each cell type population, the fold change for that cell type-specific gene (e.g. CK19 for duct cells) is graphed, compared to the transcript level for that gene in the total pancreas. Thus, for example the CK19 value for PNA-sorted pancreas is roughly 17% of the CK19 for the whole pancreas. If we estimate that 2% of the whole pancreas typically contains ducts, then only 17% of those 2%, or 0.34% of the cells in the PNA-sorted preparation are contaminating duct cells.

**Figure 3:**
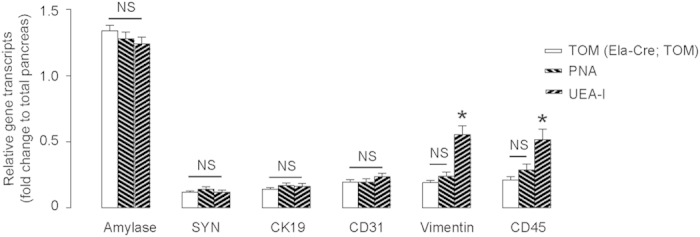
PNA-purified acinar cells contain fewer contaminating mesenchymal and inflammatory cells. The purity of acinar cells that were FAC sorted using UEA-I or PNA from C57BL/6 mice, or by tomato from Ela-Cre; TOM mice was compared to the unlabeled dissociated pancreatic cells from C57BL/6 mice by examination of mRNA levels of specific cell marker genes, including amylase (for acinar cells), SYN (for endocrine cells), CK19 (for duct cells), CD31 (for endothelial cells), vimentin (for mesenchymal cells), and CD45 (for inflammatory cells). *p < 0.05. NS: non-significant. N = 5.

We found that the purity of acinar cells sorted by PNA was equivalent to that of Ela-Cre; TOM mice (Fig. 3), suggesting that PNA did not have significant affinity to non-acinar cells in the mouse pancreas, and did have a specific affinity to acinar cells, with the specificity comparable to transgenic mice with an acinar-cell-specific fluorescent reporter. On the other hand, although UEA-I-purified acinar cells had a similar purity with regard to transcripts encoding amylase, SYN, CK19 and CD31, compared with PNA, they had significantly higher levels of transcripts for vimentin and CD45 (Fig. 3), suggesting that UEA-I may have a higher affinity for mesenchymal and inflammatory cells, compared to PNA. Thus, in particular, under circumstances where there are increased numbers of mesenchymal cells or inflammatory cells in the pancreas, using UEA-I to purify acinar cells may result in significantly greater non-acinar contamination, compared to PNA.

### PNA-purified acinar cells from NOD mice contain fewer contaminating inflammatory cells

**Figure 4:**
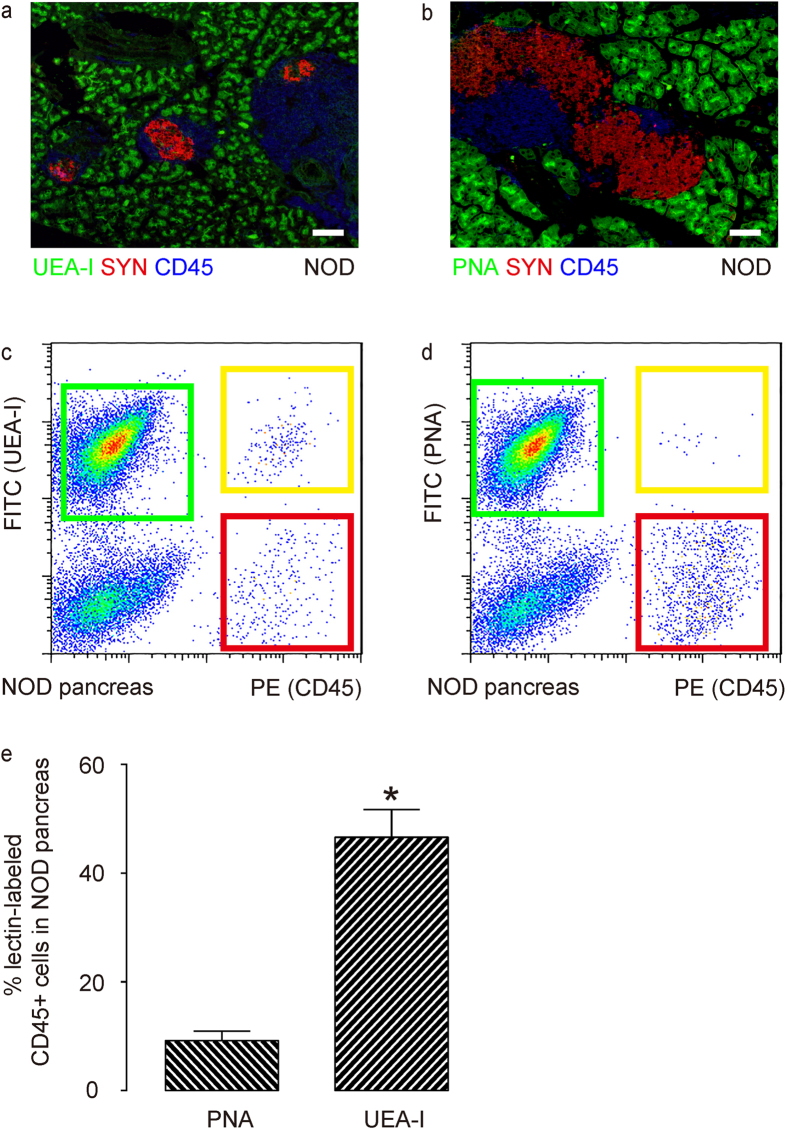
PNA-purified acinar cell populations from NOD mice contain fewer contaminating inflammatory cells. (**a,b**) UEA-I (**a**) or PNA (**b**)-labelled NOD mouse pancreas were stained with synaptophysin (SYN) and CD45. (**c,d**) Representative flow charts for UEA-I and CD45 (**c**) or PNA and CD45 (**d**)-labeled NOD mouse pancreatic digests. (**e**) Quantification of the percentage of lectin-labelled cells in all CD45-positive cells in these mice. *p < 0.05. N = 5. Scale bars are 20 μm.

To confirm the above findings, we used UEA-I (Fig. 4a) and PNA (Fig. 4b) to independently label the pancreatic cells from NOD mice that contain infiltrating inflammatory cells due to insulitis. We co-labeled the cells with fluorescence-conjugated CD45 antibody as a marker of immune cells, and analyzed the pancreatic cells by flow cytometry. We found that PNA-sorted cells from NOD mice contained markedly fewer CD45-positive inflammatory cells, compared to UEA-I-sorted cells, shown by representative flow charts (Fig. 4c,d), and by quantification (Fig. 4e). These data confirm that PNA-purified acinar cell populations are less likely to contain contaminating inflammatory cells than UEA-I-purified acinar cells.

## Discussion

The generation of functional pancreatic beta-cells to compensate for the short supply of donor human islets has long been regarded as a potential solution for the treatment of diabetes^39^. To this goal, great efforts have been made to generate functional beta-cells from non-beta cell sources, e.g. embryonic stem cells, pancreatic duct cells and acinar cells. Among these candidates, acinar cells may have some advantages: 1) absence of ethical issues related to embryonic stem cells; 2) acinar cells are abundant in the pancreas; 3) unlike duct cells, the identity of acinar cells is more easily confirmed. Indeed, although it is unlikely that acinar cells transdifferentiate into beta-like cells naturally^40^,^41^, previous studies have shown the possibility of converting acinar cells into beta-like cells *in vivo*, and *in vitro*, through either gene manipulations or application of growth hormone cocktails^18^,^19^,^20^,^21^,^22^. However, it is important that any study of acinar-to-beta cell conversion is done in a reliable and reproducible manner, including examination of purified acinar cells *in vitro*, which in turn is dependent on optimal purity of the cell population.

Very pure acinar cell populations can be isolated from genetically modified mice in which a fluorescent reporter is expressed under an acinar-cell-specific promoter, e.g. previously described Ela-Cre; TOM mouse^33^,^34^ and Ptf1a-CreERT/reporter mice^42^. However, these models suffer from two shortcomings. First, these mice could not be used in studies in which transgenic Cre expression was present in the pancreas under other promoters. Second, tamoxifen is typically used in these mice to induce nuclear translocation of Cre recombinase, but the side effects of tamoxifen are not completely clear, with specific concerns about expression of estrogen receptors in beta-cells^43^,^44^,^45^,^46^,^47^, and thus potential direct effects of tamoxifen on beta-cells.

As discussed before, early studies used lectin binding to acinar cells to purify them by flow cytometry^21^,^35^. These studies demonstrated a proof-of-principle regarding the possibility of using non-genetic methods to purify acinar cells. However, the degree of acinar cell purity was not thoroughly evaluated in these studies^21^. Here, we screened 20 widely used lectins for their binding specificity for major pancreatic cell types, and found 2 candidates, UEA-I and PNA, that had a strong affinity for acinar cells, with minimal affinity for endocrine, duct and endothelial cells. In the pancreas, the duct cell percentage and endocrine cell percentage are about 2% each, and the endothelial cell percentage is less than 5%. Thus, when total pancreas cells were used as a control for CK19, SYN and CD31 mRNA, less than 20% of the mRNA for each cell type was present in the sorted population, so less than 1% contamination for each non-acinar cell type.

UEA-I was used in previous studies for purifying acinar cells^21^,^35^. Besides acinar cells, endocrine cells, duct cells and endothelial cells, there are other cell types that represent minor populations in the normal pancreas. However, these cell populations may expand significantly under some pathological conditions, and thus their presence may affect the purity of lectin-sorted acinar cells in those conditions. Hence, we isolated acinar cells using either UEA-I or PNA lectin, as well as direct FAC sorting based on Ela-Cre; TOM mice fluorescence as a control. PNA-purified acinar cells contained less contamination by mesenchymal and inflammatory cells, compared to UEA-I purified acinar cells. Importantly, this difference in terms of contaminating inflammatory cells appeared to be quite pronounced in NOD mice, where one would expect significant increases in infiltrating leukocytes.

Here we focused on lectin-purification of acinar cells with regard to possible applications to diabetes research. However, purification of acinar cells may be an important adjunct to studies in pancreatitis and pancreatic carcinoma, since acinar cells may be involved in these diseases. Our study provides evidence for the preferred use of PNA rather than UEA-I to purify primary acinar cells from mouse pancreas, especially in diseases involving inflammation and tissue remodeling.

## Additional Information

**How to cite this article**: Xiao, X. *et al*. PNA lectin for purifying mouse acinar cells from the inflamed pancreas. *Sci. Rep.* **6**, 21127; doi: 10.1038/srep21127 (2016).

## Figures and Tables

**Table 1:** Subjective rating of lectin binding to pancreatic cell types.

Lectin Name	endocrine cell labeling	duct cell labeling	acinar cell labeling	endothelial cell labeling
PNA	NO	NO	Strong	NO
UEA I	NO	NO	Strong	NO
CON A	strong	strong	strong	strong
RCA120	Strong	Strong	Strong	Strong
SBA	NO	Strong	Strong	NO
DBA	NO	strong	NO	NO
WGA	Strong	Strong	Strong	Strong
GSL I	NO	NO	Strong	Weak
LCA	Weak	Strong	No	Strong
PHA-E	Weak	Weak	Weak	Weak
PHA-L	NO	NO	Weak	Weak
PSA	NO	Weak	NO	Strong
Su WGA	NO	NO	Weak	NO
GSL II	NO	NO	NO	Weak
DSL	NO	NO	NO	No
ECL	NO	Weak	Strong	NO
Jacalin	Strong	Weak	NO	NO
LEL	Strong	NO	NO	NO
STL	NO	NO	Strong	Weak
VVA	NO	Strong	NO	No

Subjective criteria: Strong: very bright fluorescence, easily visualized; Weak: not easy to see but still distinguishable from background; NO: no difference from background. Negative control: no lectin. Positive control: duodenum.
